# Weight and Blood‐Based Markers of Cachexia Predict Disability, Hospitalization and Worse Survival in Cancer Immunotherapy Patients

**DOI:** 10.1002/jcsm.13685

**Published:** 2025-01-16

**Authors:** Steven D. Tran, Noah J. Forrest, Vijeeth Guggilla, Geovanni M. Perottino, Jodi L. Johnson, Jeffrey Sosman, Ishan Roy, Theresa L. Walunas

**Affiliations:** ^1^ Center for Health Information Partnerships Northwestern University Feinberg School of Medicine Chicago Illinois USA; ^2^ Feinberg School of Medicine Northwestern University Chicago Illinois USA; ^3^ Robert H Lurie Comprehensive Cancer Center of Northwestern University Chicago Illinois USA; ^4^ Departments of Pathology, Dermatology and Medical Social Sciences Northwestern University Feinberg School of Medicine Chicago Illinois USA; ^5^ Department of Medicine, Division of Oncology Northwestern University Feinberg School of Medicine Chicago Illinois USA; ^6^ Shirley Ryan AbilityLab Chicago Illinois USA; ^7^ Department of Physical Medicine and Rehabilitation Northwestern University Feinberg School of Medicine Chicago Illinois USA; ^8^ Department of Medicine, Division of General Internal Medicine Northwestern University Feinberg School of Medicine Chicago Illinois USA

**Keywords:** cancer‐associated cachexia, electronic health record data, immune checkpoint inhibitors, survival analysis

## Abstract

**Background:**

Cancer‐associated cachexia can inhibit immune checkpoint inhibitor (ICI) therapy efficacy. Cachexia's effect on ICI therapy has not been studied in large cohorts of cancer patients aside from lung cancer. We studied associations between real‐world routinely collected clinical cachexia markers and disability‐free, hospitalization‐free and overall survival of cancer patients.

**Methods:**

A retrospective study was conducted of electronic health records (EHR) of patients with lung, renal cell, melanoma and other cancers treated with ICI therapy at Northwestern Medicine of Chicago, IL, United States, between March 2011 and January 2022. Weight, body mass index, absolute neutrophil and lymphocyte counts, albumin and C‐reactive protein (CRP) measures were analysed to calculate the Fearon consensus criteria for cachexia, weight loss grading system (WLGS) score, neutrophil‐lymphocyte ratio (NLR), Prognostic Nutritional Index (PNI) and modified Glasgow Prognostic Score (mGPS) at ICI therapy initiation. Kaplan–Meier and Cox proportional hazards analyses were used to determine associations between these metrics and disability‐free, hospitalization‐free and overall survival.

**Results:**

EHR analysis uncovered 3285 cancer patients on ICI therapy (54% > 65 years of age, 50.7% male, 77.7% White). At ICI therapy initiation, 1282 (39.0%) patients had cachexia (consensus criteria), 1641 (50.0%) had a WLGS score ≥ 2, 1806 (55.0%) had an NLR > 3, 1087 (33.1%) had albumin < 3.5 g/dL and 1318 (40.1%) had a PNI < 44. Missing measurements included CRP missing for 98.2% and mGPS missing for 98.6% of patients. Disability‐free (*n* = 1373), hospitalization‐free (*n* = 2374) and overall survival (*n* = 1599) events were analysed with 1‐year rates of 65% (64%–67%), 35% (34%–37%) and 65% (63%–66%), respectively. Multivariate Cox model analyses showed hazard ratios (HR) for cachexia at 1.58 (95% CI 1.38–1.80), 1.47 (95% CI 1.33–1.63) and 1.97 (95% CI 1.75–2.23) for disability, hospitalization and death, respectively. HRs for WLGS ≥ 2 were 1.45 (95% CI 1.28–1.66), 1.37 (95% CI 1.24–1.51) and 1.91 (95% CI 1.69–2.17). HRs for NLR > 3 were 1.57 (95% CI 1.35–1.83), 1.40 (95% CI 1.25–1.58) and 1.95 (95% CI 1.67–2.27). HRs for albumin < 3.5 g/dL were 1.33 (95% CI 1.15–1.54), 1.67 (95% CI 1.50–1.86) and 2.09 (95% CI 1.84–2.36). HRs for PNI < 44 were 1.60 (95% CI 1.39–1.84), 1.46 (95% CI 1.31–1.63) and 2.07 (95% CI 1.80–2.37).

**Conclusions:**

Fearon consensus criteria, WLGS, NLR, albumin and PNI were routinely collected at ICI initiation in regular clinical practice and predictive of worse disability‐free, hospitalization‐free and overall survival in cancer patients receiving ICI therapy. These routine clinical measures may aid prognostication and decision‐making in cancer patients with cachexia.

## 1 Introduction

Cancer‐associated cachexia is defined as loss of skeletal muscle mass, with or without loss of fat mass, that cannot be reversed by nutritional support [1]. The phenomenon is well documented with up to half of cancer patients developing cachexia and accounting for up to 20% of cancer deaths [2]. Half of cancer deaths are attributed to cancers highly associated with cachexia, including pulmonary, gastric, hepatic and colorectal cancers [1].

Though immune checkpoint inhibitor (ICI) therapy has efficacy in many malignancies, cachexia has been documented in many cancer patients undergoing ICI therapy and may worsen outcomes [3, 4, 5, 6, 7, 8, 9, 10, 11]. ICIs are antibodies that antagonize checkpoints of T‐cell development, enabling tumour‐reactive T‐cells to attack cancer cells [3, 4, 8, 12, 13]. There are currently two major classes of ICIs—one approved in 2011 that targets cytotoxic T‐lymphocyte antigen 4 (CTLA‐4) and the second approved in 2014 that targets programmed cell death protein 1 (PD‐1) or its counterpart, programmed cell death ligand 1 (PD‐L1). An estimated 44% of US cancer patients are eligible for ICI therapy [6, 7, 9]. However, studies have shown that cancer‐associated cachexia can inhibit the efficacy of ICI therapy and is associated with worse overall survival, time to treatment failure, objective response rate and progression‐free survival [10, 11, 14, 15, 16, 17, 18, 19, 20, 21, 22, 23, 24]. A proposed mechanism links cachexia to increased catabolic ICI clearance leading to worse survival [25, 26]. The majority of the human studies were conducted in nonsmall cell lung cancer patients, with a few studies of relatively small cohorts looking at gastric, renal cell and head and neck cancers [10, 17, 24]. The largest cohort for these prior studies included only 196 patients [15]. Most studies define cachexia by weight‐based criteria, generally weight loss greater than 5% over 6 months. Some studies include the full Fearon consensus criteria of weight loss > 2% with body mass index (BMI) < 20 kg/m^2^ [11, 14, 15, 16, 18, 19, 20, 21, 22, 23, 24]. Several studies looked at other complex markers such as skeletal muscle index, cachexia index, microbiome profiling, serum pro‐inflammatory cytokines and immune mediators, appetite‐related hormones and neutrophil‐ and platelet‐lymphocyte ratios (NLR and PLR) [16, 17, 18, 21, 22, 23, 24]. However, many of these measures and data points may not be routinely collected in current clinical practice. To our knowledge, no studies in cancer patients undergoing ICI therapy have examined emerging end points including disability‐free survival and need for hospitalization, which have been proposed to better reflect the patient experience and cachexia progression [27].

To address these gaps in knowledge, the aim of this study is to determine how common cachexia predictors (Fearon consensus criteria, weight loss grading scale [WLGS], neutrophil‐to‐lymphocyte ratio [NLR], albumin, the Prognostic Nutritional Index [PNI], C‐reactive protein [CRP] and modified Glasgow prognostic score [mGPS]) are documented in real‐world data at the point of care in the EHR and to evaluate their ability to predict disability‐free, hospitalization‐free and overall survival. These cachexia variables have all been suggested to be indicators of cancer‐associated cachexia, with a few examples in cancers treated with ICIs or other immunotherapy [17, 24, 28, 29, 30, 31, 32, 33, 34, 35], and represent a battery of routine tests in clinical care that may serve as actionable clinical criteria for cachexia interventions such as prehabilitation or nutritional therapy.

## 2 Methods

### 2.1 Patient Population

The study population included patients seen in the Robert H. Lurie Comprehensive Cancer Center at Northwestern Medicine (NM), a large healthcare system providing inpatient, outpatient and specialty care throughout Chicago and Northern Illinois. Data were acquired from the Northwestern Medicine Enterprise Data Warehouse (NMEDW), NM's clinical research database containing data on over 10 million patients. This study was governed by the Northwestern University Institutional Review Board (NU IRB), Protocol Nos. STU00210502 and STU00206779. We retrospectively identified an ICI cohort as all patients aged 18 to 99 with a diagnosis of cancer (melanoma, renal cell carcinoma, nonsmall cell and small cell lung carcinoma, urothelial cancer, head and neck cancer, gastric cancer, colon cancer, liver cancer, cervical cancer, uterine cancer, breast cancer, Hodgkin's lymphoma, Merkel cell carcinoma, rectal cancer, prostate cancer, oesophageal cancer, leukaemia or lymphoma) who received at least one dose of ICI therapy (pembrolizumab, nivolumab, cemiplimab, atezolizumab, avelumab, durvalumab, ipilimumab or tremilumumab) between 1 March 2011 and 1 January 2022. Cancer was identified in the NMEDW by International Classification of Disease‐9^th^ revision‐Clinical Modification (ICD‐9‐CM) and ICD‐10‐CM diagnosis codes. ICIs were identified in the NMEDW by a regular expression search for the generic and brand name in the medication data table (Table S1).

Sex, race and ethnicity were gathered from patient demographic data present in the NMEDW. Cancer stage and Eastern Cooperative Oncology Group Performance Status (ECOG PS) were both extracted from the oncology notes by regular expression search. We used cancer stage and ECOG PS closest to the first date of ICI infusion within 30 days. Regex search strings are in Table S1.

### 2.2 Cachexia Predictors

Cachexia predictors included the Fearon consensus criteria for cachexia (consensus criteria), weight loss grading system (WLGS), neutrophil to lymphocyte ratio (NLR), albumin, CRP, the PNI, and the mGPS. Cachexia predictors were determined at baseline, defined as the result closest to the date of ICI initiation within 14 days prior.

From the NMEDW, we extracted weight (kg) and BMI (kg/m^2^) data for the consensus criteria and WLGS. Absolute neutrophil count (ANC, cells/μl), absolute lymphocyte count (ALC, cells/μl), albumin (g/dL) and CRP (mg/L) were extracted for lab‐based cachexia measurements. Values for each lab result were normalized to the above units. For the consensus criteria and WLGS, we calculated percent weight loss at baseline compared to the peak weight in the preceding 6 months. For the consensus criteria, we defined cachexia to be > 5% weight loss or > 2% weight loss with BMI < 20 kg/m^2^ on the same date. WLGS was calculated based on the published grading scale using weight loss as described and BMI on the same date [29]. NLR was calculated using ANC and ALC data collected on the same date. PNI was calculated as 10*albumin (g/dL) + 0.005*ALC (cells/μL) using albumin and ALC on the same date [32]. mGPS was calculated using published scoring rules based on CRP and albumin values on the same date [34]. To limit model complexity in the survival analysis, NLR was dichotomized to ≤ 3 or > 3; albumin to ≥ 3.5 or < 3.5 g/dL; and PNI to ≥ 44 or < 44.

High levels of missingness for CRP and mGPS data (98.2% and 98.6% of patients missing these data, respectively) rendered us unable to use these measures for further analyses.

### 2.3 Outcomes

Primary clinical endpoints evaluated were disability‐free, hospitalization‐free and overall survival. Time to rehabilitation order, admission and death were calculated from the date of ICI initiation to the date of rehabilitation order (defined by the first date of an order for rehabilitation services such as physical or occupational therapy), admission (defined as the first date of any emergency department or inpatient encounter) and death, respectively.

### 2.4 Survival Analysis: Kaplan–Meier

Kaplan–Meier analysis was performed for each cachexia predictor (consensus criteria, WLGS, NLR, albumin and PNI) and outcome pair (disability‐free, hospitalization‐free and overall survival). Analysis was done in the total ICI cohort as well as for lung cancer, renal cell cancer and melanoma—the three most common ICI‐sensitive cancers. Patients were censored for disability‐ and hospitalization‐free survival based on the last follow‐up date (or death), defined as the last date of in‐person or telehealth visit. The log‐rank test was used to determine statistical significance at the 95% confidence level.

### 2.5 Survival Analysis: Cox Proportional Hazard Model

Cox proportional hazard models were used to evaluate hazard ratios (HRs) for each clinical endpoint. For each cachexia predictor, we calculated an unadjusted model and a model adjusting for age, sex, race, ethnicity, cancer type, first ICI class, cancer stage and ECOG. We dichotomized all input variables for use in the Cox models. Age was dichotomized to ≤ 65 years (reference) and > 65 years; sex to female (reference) and male; race to White (reference) and non‐White; ethnicity to Hispanic or Latino (reference) and non‐Hispanic or Latino; cancer to lung cancer (reference) and non‐lung cancer; ICI to PD‐1/L1 (reference) and CTLA‐4 or combination PD‐1/CTLA‐4 therapy; ECOG to 0 (reference) and > 0; stage to I–III (reference) and IV; consensus criteria to no cachexia (reference) and cachexia (> 5% weight loss or > 2% weight loss and BMI < 20); WLGS to 0–1 (reference) and 2–4; NLR to ≤ 3 (reference) and > 3; albumin to > = 3.5 (reference) and < 3.5; and PNI to ≥ 44 (reference) and < 44. Cox model analysis was conducted for the total ICI cohort as well as for lung and renal cell cancers and melanoma.

For each model, we tested for the proportional hazards assumption by visualizing scaled Schoenfeld residuals versus time as well as for influential/outlier observations by visualizing change in regression coefficients upon removal of each observation.

Kaplan–Meier curves and Cox proportional hazard models were developed using the *survival* and *survminer* packages in R 4.3.2 [36].

## 3 Results

### 3.1 Cohort Description

There were 3285 cancer patients who received ICI therapy between 1 March 2011 and 1 January 2022 in our EHR analysis. Patient characteristics are described in Table 1. Of those, 50.7% were male, 77.7% were White, 89.5% were non‐Hispanic or Latino and 54.0% were at least 65 years of age at the time of cancer diagnosis. The most common cancers were lung (*N* = 1266 [38.5%]), melanoma (*N* = 477 [14.5%]) and renal (*N* = 329 [10.0%]). Pembrolizumab was the most common first ICI regimen (*N* = 1399 [42.6%]) followed by nivolumab (*N* = 817 [24.9%]) and combined nivolumab + ipilimumab (*N* = 402 [12.2%]).

**TABLE 1:** Basic demographics, cancer and ICI characteristics, and cachexia predictors—stratified by cancer type.

	Overall	Lung cancer	Kidney cancer	Melanoma	Other cancer
	(***N*** = 3285)	(*N* = 1266)	(*N* = 329)	(*N* = 477)	(***N*** = 1213)
Sex, *N* (%)					
Female	1621 (49.3%)	653 (51.6%)	109 (33.1%)	193 (40.5%)	666 (54.9%)
Male	1664 (50.7%)	613 (48.4%)	220 (66.9%)	284 (59.5%)	547 (45.1%)
Race, *N* (%)					
American Indian or Alaska Native	8 (0.2%)	3 (0.2%)	2 (0.6%)	0 (0%)	3 (0.2%)
Asian	144 (4.4%)	58 (4.6%)	16 (4.9%)	6 (1.3%)	64 (5.3%)
Black or African American	279 (8.5%)	153 (12.1%)	20 (6.1%)	3 (0.6%)	103 (8.5%)
Native Hawaiian or Other Pacific Islander	6 (0.2%)	2 (0.2%)	2 (0.6%)	0 (0%)	2 (0.2%)
White	2553 (77.7%)	960 (75.8%)	243 (73.9%)	421 (88.3%)	929 (76.6%)
Other	41 (1.2%)	15 (1.2%)	3 (0.9%)	5 (1.0%)	18 (1.5%)
Unknown	254 (7.7%)	75 (5.9%)	43 (13.1%)	42 (8.8%)	94 (7.7%)
Ethnicity, *N* (%)					
Hispanic or Latino	176 (5.4%)	45 (3.6%)	30 (9.1%)	13 (2.7%)	88 (7.3%)
Not Hispanic or Latino	2939 (89.5%)	1164 (91.9%)	274 (83.3%)	433 (90.8%)	1068 (88.0%)
Unknown	170 (5.2%)	57 (4.5%)	25 (7.6%)	31 (6.5%)	57 (4.7%)
Age, *N* (%)					
< 65	1511 (46.0%)	459 (36.3%)	155 (47.1%)	274 (57.4%)	623 (51.4%)
≥ 65	1774 (54.0%)	807 (63.7%)	174 (52.9%)	203 (42.6%)	590 (48.6%)
Cancer type, *N* (%)					
Lung cancer	1266 (38.5%)	1266 (100%)	0 (0%)	0 (0%)	0 (0%)
Melanoma	477 (14.5%)	0 (0%)	0 (0%)	477 (100%)	0 (0%)
Kidney cancer	329 (10.0%)	0 (0%)	329 (100%)	0 (0%)	0 (0%)
Other cancer	1213 (36.9%)	0 (0%)	0 (0%)	0 (0%)	1213 (100%)
Cancer stage, *N* (%)					
I	253 (7.7%)	94 (7.4%)	13 (4.0%)	32 (6.7%)	114 (9.4%)
II	241 (7.3%)	52 (4.1%)	12 (3.6%)	60 (12.6%)	117 (9.6%)
III	649 (19.8%)	231 (18.2%)	43 (13.1%)	185 (38.8%)	190 (15.7%)
IV	1301 (39.6%)	645 (50.9%)	164 (49.8%)	96 (20.1%)	396 (32.6%)
Missing	841 (25.6%)	244 (19.3%)	97 (29.5%)	104 (21.8%)	396 (32.6%)
ECOG, *N* (%)					
0	1096 (33.4%)	267 (21.1%)	153 (46.5%)	253 (53.0%)	423 (34.9%)
1	1378 (41.9%)	611 (48.3%)	106 (32.2%)	146 (30.6%)	515 (42.5%)
2	441 (13.4%)	247 (19.5%)	39 (11.9%)	25 (5.2%)	130 (10.7%)
3	81 (2.5%)	37 (2.9%)	10 (3.0%)	8 (1.7%)	26 (2.1%)
4	4 (0.1%)	0 (0%)	1 (0.3%)	1 (0.2%)	2 (0.2%)
Missing	285 (8.7%)	104 (8.2%)	20 (6.1%)	44 (9.2%)	117 (9.6%)
ICI, *N* (%)					
Pembrolizumab	1399 (42.6%)	569 (44.9%)	76 (23.1%)	104 (21.8%)	650 (53.6%)
Nivolumab	817 (24.9%)	262 (20.7%)	138 (41.9%)	154 (32.3%)	263 (21.7%)
Nivolumab/Ipilimumab	402 (12.2%)	92 (7.3%)	95 (28.9%)	119 (24.9%)	96 (7.9%)
Atezolizumab	339 (10.3%)	205 (16.2%)	14 (4.3%)	3 (0.6%)	117 (9.6%)
Durvalumab	188 (5.7%)	134 (10.6%)	2 (0.6%)	1 (0.2%)	51 (4.2%)
Ipilimumab	116 (3.5%)	3 (0.2%)	1 (0.3%)	96 (20.1%)	16 (1.3%)
Avelumab	22 (0.7%)	0 (0%)	3 (0.9%)	0 (0%)	19 (1.6%)
Cemiplimab	2 (0.1%)	1 (0.1%)	0 (0%)	0 (0%)	1 (0.1%)
Weight‐based cachexia, *N* (%)					
No cachexia	1938 (59.0%)	701 (55.4%)	205 (62.3%)	367 (76.9%)	665 (54.8%)
Cachexia	1282 (39.0%)	539 (42.6%)	121 (36.8%)	102 (21.4%)	520 (42.9%)
Missing	65 (2.0%)	26 (2.1%)	3 (0.9%)	8 (1.7%)	28 (2.3%)
Weight loss grading system (WLGS), *N* (%)					
0	937 (28.5%)	287 (22.7%)	129 (39.2%)	212 (44.4%)	309 (25.5%)
1	642 (19.5%)	243 (19.2%)	48 (14.6%)	112 (23.5%)	239 (19.7%)
2	629 (19.1%)	266 (21.0%)	53 (16.1%)	72 (15.1%)	238 (19.6%)
3	662 (20.2%)	285 (22.5%)	59 (17.9%)	59 (12.4%)	259 (21.4%)
4	350 (10.7%)	159 (12.6%)	37 (11.2%)	14 (2.9%)	140 (11.5%)
Missing	65 (2.0%)	26 (2.1%)	3 (0.9%)	8 (1.7%)	28 (2.3%)
Neutrophil‐to‐lymphocyte ratio, *N* (%)					
NLR ≤ 3	895 (27.2%)	262 (20.7%)	107 (32.5%)	182 (38.2%)	344 (28.4%)
NLR > 3	1806 (55.0%)	825 (65.2%)	148 (45.0%)	156 (32.7%)	677 (55.8%)
Missing	584 (17.8%)	179 (14.1%)	74 (22.5%)	139 (29.1%)	192 (15.8%)
Albumin (g/dL), *N* (%)					
Albumin ≥ 3.5	2138 (65.1%)	844 (66.7%)	205 (62.3%)	353 (74.0%)	736 (60.7%)
Albumin < 3.5	1087 (33.1%)	401 (31.7%)	119 (36.2%)	118 (24.7%)	449 (37.0%)
Missing	60 (1.8%)	21 (1.7%)	5 (1.5%)	6 (1.3%)	28 (2.3%)
C‐reactive protein (mg/L), *N* (%)					
CRP > 3	56 (1.7%)	24 (1.9%)	6 (1.8%)	7 (1.5%)	19 (1.6%)
CRP ≤ 3	2 (0.1%)	1 (0.1%)	0 (0%)	1 (0.2%)	0 (0%)
Missing	3227 (98.2%)	1241 (98.0%)	323 (98.2%)	469 (98.3%)	1194 (98.4%)
Prognostic Nutritional Index, *N* (%)					
PNI ≥ 44	1347 (41.0%)	513 (40.5%)	138 (41.9%)	241 (50.5%)	455 (37.5%)
PNI < 44	1318 (40.1%)	559 (44.2%)	115 (35.0%)	92 (19.3%)	552 (45.5%)
Missing	620 (18.9%)	194 (15.3%)	76 (23.1%)	144 (30.2%)	206 (17.0%)
Modified Glasgow Prognostic Score, *N* (%)					
0	13 (0.4%)	8 (0.6%)	0 (0%)	3 (0.6%)	2 (0.2%)
1	17 (0.5%)	5 (0.4%)	3 (0.9%)	3 (0.6%)	6 (0.5%)
2	17 (0.5%)	5 (0.4%)	2 (0.6%)	1 (0.2%)	9 (0.7%)
Missing	3238 (98.6%)	1248 (98.6%)	324 (98.5%)	470 (98.5%)	1196 (98.6%)

At baseline, within 14 days prior ICI initiation, 1282 (39.0%) patients had cachexia as defined by the Fearon consensus criteria, 1641 (50.0%) patients had a WLGS score ≥ 2, 1806 (55.0%) patients had an NLR > 3, 1087 (33.1%) patients had an albumin < 3.5 g/dL, and 1318 (40.1%) patients had a PNI < 44. There were 65 (2.0%) patients missing consensus criteria data, 65 (2.0%) patients missing WLGS data, 584 (17.8%) patients missing NLR data, 60 (1.8%) patients missing albumin data and 620 (18.9%) patients missing PNI data. Patients missing consensus criteria, WLGS, NLR, albumin and PNI data were excluded from the respective survival analyses. CRP and mGPS were excluded from survival analyses due to high missingness (98.2% and 98.6% missingness, respectively). Median follow‐up time was 420.5 days in the overall cohort, 342.5 days in the lung cancer cohort, 654 days in the renal cell cancer cohort and 727 days in the melanoma cohort.

### 3.2 Outcomes Assessment Overall and by Cancer Type

The overall cohort had 1373 total rehabilitation order events. The 1‐year disability‐free survival rate was 65% (95% CI 64%–67%), and the 3‐year disability‐free survival rate was 44% (95% CI 42%–47%) in the overall ICI cohort (Table 2). The 1‐year disability‐free survival rate was 58% (95% CI 54%–61%), and the 3‐year disability‐free survival rate was 32% (95% CI 28%–36%) in the lung cancer cohort. The 1‐year disability‐free survival rate was 71% (95% CI 65%–76%), and the 3‐year disability‐free survival rate was 45% (95% CI 38%–52%) in the renal cell cancer cohort. The 1‐year disability‐free survival rate was 79% (95% CI 76%–83%), and the 3‐year disability‐free survival rate was 65% (95% CI 60%–70%) in the melanoma cohort.

**TABLE 2:** 1‐ and 3‐year survival rates for three clinical endpoints—disability‐free, hospitalization‐free and overall survival—stratified by cachexia predictor.

Disability‐free survival								
	Overall		Lung cancer		Renal cell cancer		Melanoma	
Cachexia predictor	1‐year disability‐free survival rate (95% CI)	3‐year disability‐free survival rate (95% CI)	1‐year disability‐free survival rate (95% CI)	3‐year disability‐free survival rate (95% CI)	1‐year disability‐free survival rate (95% CI)	3‐year disability‐free survival rate (95% CI)	1‐year disability‐free survival rate (95% CI)	3‐year disability‐free survival rate (95% CI)
Overall	65% (64%–67%)	44% (42%–47%)	58% (54%–61%)	32% (28%–36%)	71% (65%–76%)	45% (38%–52%)	79% (76%–83%)	65% (60%–70%)
Fearon Consensus Criteria								
No cachexia	71% (69%–74%)	51% (48%–54%)	62% (58%–66%)	35% (30%–40%)	76% (70%–82%)	52% (44%–61%)	84% (80%–88%)	69% (64%–75%)
Cachexia	55% (52%–58%)	32% (29%–37%)	51% (46%–56%)	26% (21%–33%)	59% (50%–71%)	29% (20%–44%)	61% (51%–73%)	47% (36%–63%)
Weight loss grading system (WLGS)								
WLGS = 0	74% (71%–77%)	52% (48%–56%)	63% (57%–69%)	30% (23%–38%)	81% (74%–88%)	55% (45%–67%)	87% (82%–92%)	74% (67%–82%)
WLGS = 1	70% (67%–74%)	50% (45%–56%)	66% (59%–73%)	44% (36%–53%)	71% (59%–87%)	41% (26%–65%)	76% (68%–85%)	59% (49%–71%)
WLGS = 2	57% (53%–62%)	39% (34%–45%)	49% (42%–56%)	27% (20%–36%)	60% (47%–76%)	34% (19%–60%)	71% (61%–83%)	53% (41%–69%)
WLGS = 3	59% (55%–64%)	37% (32%–43%)	58% (51%–65%)	31% (24%–41%)	60% (47%–75%)	36% (23%–57%)	68% (56%–84%)	59% (45%–77%)
WLGS = 4	54% (48%–61%)	30% (23%–41%)	49% (40%–60%)	30% (20%–44%)	57% (39%–84%)	29% (12%–70%)	81% (60%–100%)	81% (60%–100%)
Neutrophil‐to‐lymphocyte ratio (NLR)								
NLR ≤ 3	74% (72%–78%)	52% (48%–56%)	63% (57%–70%)	37% (30%–46%)	85% (78%–92%)	53% (43%–67%)	86% (81%–91%)	70% (63%–78%)
NLR > 3	53% (51%–56%)	33% (30%–37%)	50% (47%–54%)	25% (21%–30%)	50% (41%–60%)	29% (21%–41%)	63% (55%–72%)	52% (43%–62%)
Albumin (g/dL)								
Albumin ≥ 3.5	70% (67%–72%)	48% (45%–50%)	59% (56%–63%)	33% (29%–38%)	77% (71%–83%)	47% (39%–57%)	85% (81%–89%)	69% (63%–74%)
Albumin < 3.5	56% (53%–60%)	38% (33%–43%)	56% (50%–62%)	26% (18%–36%)	58% (48%–70%)	39% (29%–54%)	61% (51%–72%)	58% (48%–71%)
Prognostic Nutritional Index (PNI)								
PNI ≥ 44	72% (69%–74%)	50% (46%–53%)	61% (57%–66%)	36% (31%–42%)	78% (71%–85%)	48% (39%–60%)	84% (80%–89%)	69% (62%–76%)
PNI < 44	48% (45%–52%)	29% (25%–32%)	45% (40%–50%)	20% (15%–26%)	49% (39%–60%)	27% (18%–41%)	52% (41%–64%)	42% (29%–59%)

Hospitalization‐free survival								
	Overall		Lung cancer		Renal cell cancer		Melanoma	
Cachexia predictor	1‐year hospitalization‐free survival rate (95% CI)	3‐year hospitalization‐free survival rate (95% CI)	1‐year hospitalization‐free survival rate (95% CI)	3‐year hospitalization‐free survival rate (95% CI)	1‐year hospitalization‐free survival rate (95% CI)	3‐year hospitalization‐free survival rate (95% CI)	1‐year hospitalization‐free survival rate (95% CI)	3‐year hospitalization‐free survival rate (95% CI)
Overall	35% (34%–37%)	18% (17%–20%)	31% (28%–34%)	13% (10%–15%)	43% (38%–49%)	21% (16%–27%)	46% (42%–51%)	29% (25%–34%)
Fearon Consensus Criteria								
No cachexia	42% (40%–44%)	23% (21%–25%)	35% (31%–39%)	15% (12%–19%)	51% (44%–59%)	27% (20%–36%)	52% (47%–57%)	34% (29%–40%)
Cachexia	25% (23%–28%)	10% (8.4%–13%)	25% (21%–29%)	9.5% (6.7%–14%)	29% (21%–39%)	9.6% (4.7%–20%)	23% (16%–34%)	9.6% (4.5%–20%)
Weight loss grading system (WLGS)								
WLGS = 0	44% (41%–48%)	25% (22%–29%)	36% (31%–42%)	13% (9.4%–19%)	56% (48%–66%)	30% (22%–42%)	52% (45%–59%)	35% (29%–43%)
WLGS = 1	41% (37%–46%)	19% (16%–24%)	36% (30%–44%)	14% (9.1%–21%)	46% (34%–63%)	14% (5.3%–36%)	56% (48%–67%)	35% (26%–46%)
WLGS = 2	32% (28%–36%)	18% (15%–23%)	25% (20%–31%)	15% (10%–21%)	37% (25%–53%)	17% (7.2%–38%)	33% (23%–46%)	23% (15%–36%)
WLGS = 3	28% (25%–33%)	12% (8.7%–15%)	30% (25%–37%)	12% (7.5%–18%)	28% (18%–44%)	13% (5.5%–31%)	27% (17%–41%)	14% (7.0%–30%)
WLGS = 4	20% (16%–25%)	7.6% (4.5%–13%)	22% (16%–31%)	7.7% (2.9%–20%)	24% (12%–45%)	9.9% (2.9%–34%)	21% (6.5%–64%)	
Neutrophil‐to‐lymphocyte ratio (NLR)								
NLR ≤ 3	47% (44%–51%)	27% (24%–31%)	37% (32%–44%)	19% (14%–25%)	64% (56%–74%)	33% (24%–46%)	59% (52%–67%)	39% (31%–47%)
NLR > 3	30% (28%–33%)	13% (11%–16%)	30% (27%–33%)	10% (7.8%–14%)	30% (23%–40%)	9.9% (4.9%–20%)	36% (29%–45%)	24% (17%–33%)
Albumin (g/dL)								
Albumin ≥ 3.5	43% (40%–45%)	22% (20%–24%)	36% (32%–39%)	16% (13%–19%)	54% (47%–61%)	23% (17%–32%)	52% (47%–58%)	33% (28%–39%)
Albumin < 3.5	20% (17%–23%)	10% (8.1%–13%)	20% (16%–25%)	5.6% (3.1%–10%)	23% (16%–33%)	15% (8.9%–25%)	25% (17%–35%)	19% (13%–29%)
Prognostic Nutritional Index (PNI)								
PNI ≥ 44	46% (43%–49%)	23% (21%–26%)	40% (36%–45%)	17% (13%–21%)	55% (47%–64%)	24% (16%–35%)	57% (51%–63%)	37% (30%–44%)
PNI < 44	26% (23%–28%)	13% (11%–16%)	24% (20%–28%)	8.6% (5.8%–13%)	33% (25%–44%)	17% (11%–28%)	26% (18%–38%)	21% (13%–32%)

Overall survival								
	Overall		Lung cancer		Renal cell cancer		Melanoma	
Cachexia predictor	1‐year survival rate (95% CI)	3‐year survival rate (95% CI)	1‐year survival rate (95% CI)	3‐year survival rate (95% CI)	1‐year survival rate (95% CI)	3‐year survival rate (95% CI)	1‐year survival rate (95% CI)	3‐year survival rate (95% CI)
Overall	65% (63%–66%)	44% (43%–47%)	58% (55%–61%)	34% (31%–37%)	73% (68%–78%)	55% (49%–62%)	79% (75%–83%)	66% (61%–71%)
Fearon Consensus Criteria								
No cachexia	75% (73%–77%)	55% (52%–57%)	68% (64%–72%)	42% (38%–46%)	84% (79%–89%)	65% (58%–74%)	84% (81%–88%)	73% (68%–78%)
Cachexia	48% (45%–51%)	29% (26%–32%)	44% (40%–49%)	24% (21%–29%)	53% (44%–63%)	35% (26%–46%)	57% (47%–69%)	39% (29%–53%)
Weight loss grading system (WLGS)								
WLGS = 0	80% (78%–83%)	59% (55%–63%)	76% (71%–81%)	46% (39%–53%)	87% (81%–93%)	70% (61%–80%)	87% (83%–92%)	78% (72%–84%)
WLGS = 1	73% (70%–77%)	53% (48%–58%)	65% (59%–71%)	41% (34%–49%)	79% (68%–91%)	54% (38%–75%)	81% (73%–89%)	66% (57%–76%)
WLGS = 2	62% (58%–66%)	40% (36%–45%)	58% (52%–65%)	33% (27%–41%)	68% (57%–83%)	50% (37%–68%)	70% (60%–82%)	52% (40%–67%)
WLGS = 3	50% (46%–55%)	33% (29%–37%)	46% (40%–52%)	28% (22%–35%)	64% (52%–78%)	46% (33%–63%)	61% (49%–76%)	46% (33%–63%)
WLGS = 4	35% (30%–41%)	18% (14%–25%)	34% (27%–43%)	14% (8.3%–25%)	31% (18%–53%)	12% (4.1%–33%)	39% (18%–86%)	39% (18%–86%)
Neutrophil‐to‐lymphocyte ratio (NLR)								
NLR ≤ 3	82% (79%–85%)	63% (59%–67%)	74% (68%–79%)	49% (42%–57%)	90% (84%–96%)	76% (67%–86%)	91% (86%–95%)	83% (77%–89%)
NLR > 3	55% (53%–57%)	35% (32%–38%)	53% (49%–56%)	30% (27%–34%)	58% (50%–67%)	37% (28%–48%)	69% (62%–77%)	54% (46%–64%)
Albumin (g/dL)								
Albumin ≥ 3.5	74% (72%–76%)	53% (50%–55%)	65% (62%–69%)	40% (36%–44%)	84% (79%–89%)	67% (59%–75%)	85% (82%–89%)	74% (69%–79%)
Albumin < 3.5	45% (42%–49%)	28% (25%–31%)	42% (37%–47%)	23% (19%–29%)	53% (44%–63%)	33% (25%–45%)	59% (50%–69%)	39% (30%–51%)
Prognostic Nutritional Index (PNI)								
PNI ≥ 44	79% (77%–81%)	59% (56%–62%)	70% (66%–75%)	45% (41%–51%)	85% (80%–92%)	72% (63%–81%)	89% (85%–93%)	79% (74%–85%)
PNI < 44	49% (46%–52%)	29% (26%–33%)	46% (42%–51%)	25% (21%–30%)	55% (46%–65%)	31% (22%–44%)	60% (51%–72%)	45% (35%–58%)

The overall cohort had 2374 total emergency or inpatient admission events. The 1‐year hospitalization‐free survival rate was 35% (95% CI 34%–37%), and the 3‐year hospitalization‐free survival rate was 18% (95% CI 17%–20%) in the overall ICI cohort (Table 2). The 1‐year hospitalization‐free survival rate was 31% (95% CI 28%–34%), and the 3‐year hospitalization‐free survival rate was 13% (95% CI 10%–15%) in the lung cancer cohort. The 1‐year hospitalization‐free survival rate was 43% (95% CI 38%–49%), and the 3‐year hospitalization‐free survival rate was 21% (95% CI 16%–27%) in the renal cell cancer cohort. The 1‐year hospitalization‐free survival rate was 46% (95% CI 42%–51%), and the 3‐year hospitalization‐free survival rate was 29% (95% CI 25%–34%) in the melanoma cohort.

For overall survival, the cohort had 1599 total deaths. The 1‐year overall survival rate was 65% (95% CI 63%–66%), and the 3‐year overall survival rate was 44% (95% CI 43%–47%) in the overall ICI cohort (Table 2). The 1‐year overall survival rate was 58% (95% CI 55%–61%), and the 3‐year overall survival rate was 34% (95% CI 31%–37%) in the lung cancer cohort. The 1‐year overall survival rate was 73% (95% CI 68%–78%), and the 3‐year overall survival rate was 55% (95% CI 49%–62%) in the renal cell cancer cohort. The 1‐year overall survival rate was 79% (95% CI 75%–83%), and the 3‐year overall survival rate was 66% (95% CI 61%–71%) in the melanoma cohort.

### 3.3 Weight Loss, WLGS, NLR, Albumin and PNI Are Associated With Worse Outcomes for Immunotherapy Patients as Assessed in Kaplan–Meier Analysis

**FIGURE 1:**
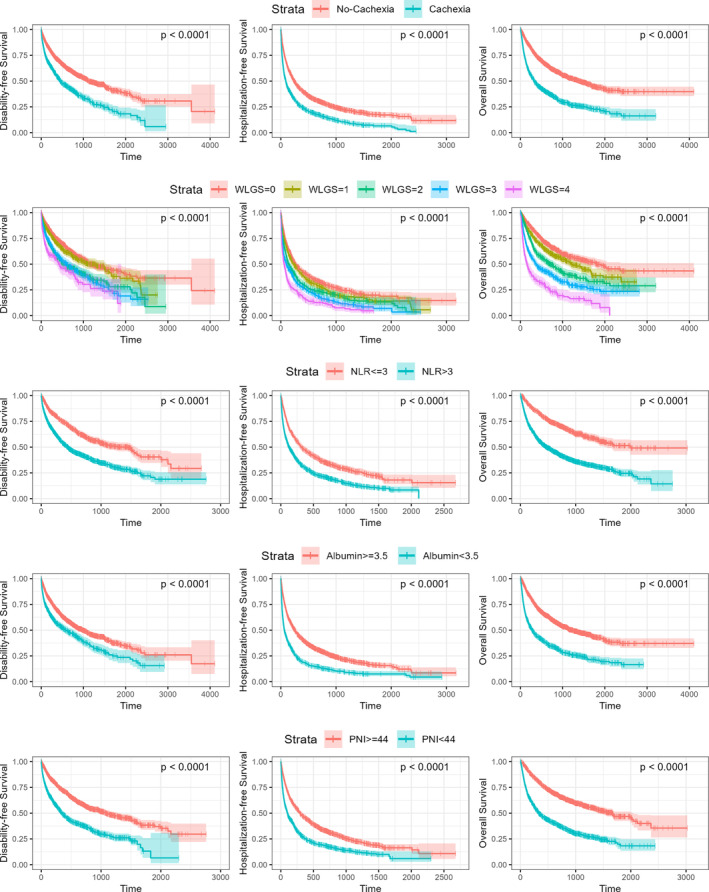
Kaplan–Meier survival curves in the total ICI cohort for the cachexia predictors—Fearon consensus criteria for cachexia, WLGS, NLR, albumin and PNI—compared to the clinical endpoints—disability‐free, hospitalization‐free and overall survival. *p* values were calculated by log‐rank test.

Kaplan–Meier survival curves are shown in Figure 1 and the 1‐ and 3‐year disability‐free, hospitalization‐free and overall survival rates are given in Table 2. Patients with cachexia (based on the Fearon consensus criteria) had significantly lower disability‐free, hospitalization‐free and overall survival rates compared to patients with no cachexia. The 1‐year disability‐free survival rate (no cachexia vs. cachexia) was 71% (95% CI 69%–74%) versus 55% (95% CI 52%–58%). The 1‐year hospitalization‐free survival rate was 42% (95% CI 40%–44%) versus 25% (95% CI 23%–28%). The 1‐year overall survival rate was 75% (95% CI 73–77%) versus 48% (95% CI 46%–51%).

WLGS also differentiated between the time to each endpoint, with a score of 4 being associated with lower disability‐free, hospitalization‐free and overall survival rates compared to a score of 0. The 1‐year disability‐free survival rate (WLGS = 0 vs. WLGS = 4) was 74% (95% CI 72%–78%) versus 54% (95% CI 48%–61%). The 1‐year hospitalization‐free survival rate was 44% (95% CI 41%–48%) versus 20% (95% CI 16%–25%). The 1‐year overall survival rate was 81% (95% CI 78–83%) versus 35% (95% CI 30–41%).

NLR > 3 was associated with lower disability‐free, hospitalization‐free and overall survival rates. The 1‐year disability‐free survival rate (NLR ≤ 3 vs. NLR > 3) was 75% (95% CI 72%–78%) versus 54% (95% CI 51%–56%). The 1‐year hospitalization‐free survival rate was 48% (95% CI 44%–51%) versus 30% (95% CI 28%–33%). The 1‐year survival rate was 82% (95% CI 79–84%) versus 54% (95% CI 52–57%).

Albumin < 3.5 g/dL was associated with lower disability‐free, hospitalization‐free and overall survival rates. The 1‐year disability‐free survival rate (albumin ≥ 3.5 g/dL vs. albumin < 3.5 g/dL) was 70% (95% CI 67%–72%) versus 56% (95% CI 53%–60%). The 1‐year hospitalization‐free survival rate was 43% (95% CI 40%–45%) versus 20% (95% CI 17%–23%). The 1‐year survival rate was 74% (95% CI 72%–76%) versus 45% (95% CI 42%–49%).

PNI < 44 was associated with lower disability‐free, hospitalization‐free and overall survival rates. The 1‐year disability‐free survival rate (PNI ≥ 44 vs. PNI < 44) was 72% (95% CI 69%–74%) versus 48% (95% CI 45%–52%). The 1‐year hospitalization‐free survival rate was 46% (95% CI 43%–49%) versus 26% (95% CI 23%–28%). The 1‐year survival rate was 79% (95% CI 77%–81%) versus 49% (95% CI 46%–52%).

Similar patterns were found in lung cancer, renal cell cancer and melanoma with cachexia, WLGS of 4, NLR > 3, albumin <3.5 g/dL and PNI < 44 being associated with lower disability‐free, hospitalization‐free and overall survival rates (Table 2 and Figures S1–S3). *p* values for all Kaplan–Meier curves were < 0.005.

### 3.4 Weight Loss, WLGS, NLR, Albumin and PNI Are Predictors for Increased Risk of Negative Outcomes for Immunotherapy Patients in Cox Proportional Hazards Modelling

In unadjusted Cox proportional hazards models, cachexia (Fearon consensus criteria, no cachexia as reference), WLGS ≥ 2 (WLGS < 2 reference), NLR > 3 (NLR ≤ 3 reference), albumin < 3.5 g/dL (albumin ≥ 3.5 g/dL reference) and PNI < 44 (PNI ≥ 44 reference) were all associated with shorter disability‐free, hospitalization‐free and overall survival in the overall ICI cohort (Table 3). The associations were maintained when adjusting for age, sex, race, ethnicity, cancer type, first ICI regimen, cancer stage and ECOG. Adjusted HRs for cachexia were 1.58 (95% CI 1.38–1.80), 1.47 (95% CI 1.33–1.63) and 1.97 (95% CI 1.75–2.23) for shorter disability‐free, hospitalization‐free and overall survival, respectively. HRs for WLGS ≥ 2 were 1.45 (95% CI 1.28–1.66), 1.37 (95% CI 1.24–1.51) and 1.91 (95% CI 1.69–2.17) for shorter disability‐free, hospitalization‐free and overall survival, respectively. HRs for NLR > 3 were 1.57 (95% CI 1.35–1.83), 1.40 (95% CI 1.25–1.58) and 1.95 (95% CI 1.67–2.27) for shorter disability‐free, hospitalization‐free and overall survival, respectively. HRs for albumin < 3.5 g/dL were 1.33 (95% CI 1.15–1.54), 1.67 (95% CI 1.50–1.86) and 2.09 (95% CI 1.84–2.36) for shorter disability‐free, hospitalization‐free and overall survival, respectively. HRs for PNI < 44 were 1.60 (95% CI 1.39–1.84), 1.46 (95% CI 1.31–1.63) and 2.07 (95% CI 1.80–2.37) for shorter disability‐free, hospitalization‐free and overall survival, respectively.

**TABLE 3:** Hazard ratios and *p* values in Cox proportional hazard models for three clinical endpoints—overall survival, time to rehabilitation order and time to emergency or inpatient admission—with cachexia predictors as variables. Unadjusted models included only individual cachexia predictors. Adjusted models included age, sex, race, ethnicity, cancer type, first ICI regimen, cancer stage, and ECOG.

Overall					
Cachexia predictor		Unadjusted HR	*p* value	Adjusted HR	*p* value
Disability‐free survival					
Fearon criteria‐based cachexia	No cachexia	1 (ref)		1 (ref)	
	Cachexia	1.83 (1.64–2.04)	**1.05E−27**	1.58 (1.38–1.80)	**1.54E−11**
WLGS	< 2	1 (ref)		1 (ref)	
	≥ 2	1.70 (1.52–1.89)	**9.62E−22**	1.45 (1.28–1.66)	**2.04E−08**
NLR	≤ 3	1 (ref)		1 (ref)	
	> 3	1.89 (1.67–2.13)	**1.10E−23**	1.57 (1.35–1.83)	**4.45E−09**
Albumin	≥ 3.5 g/dL	1 (ref)		1 (ref)	
	< 3.5 g/dL	1.58 (1.41–1.77)	**7.01E−15**	1.33 (1.15–1.54)	**0.00011**
PNI	≥ 44	1 (ref)		1 (ref)	
	< 44	2.02 (1.80–2.27)	**1.32E−33**	1.60 (1.39–1.84)	**6.00E−11**
Hospitalization‐free survival					
Fearon criteria‐based cachexia	No cachexia	1 (ref)		1 (ref)	
	Cachexia	1.67 (1.54–1.82)	**3.73E−34**	1.47 (1.33–1.63)	**7.48E−14**
WLGS	< 2	1 (ref)		1 (ref)	
	≥ 2	1.54 (1.42–1.67)	**2.57E−25**	1.37 (1.24–1.51)	**6.88E−10**
NLR	≤ 3	1 (ref)		1 (ref)	
	> 3	1.65 (1.49–1.81)	**7.21E−24**	1.40 (1.25–1.58)	**1.80E−08**
Albumin	≥ 3.5 g/dL	1 (ref)		1 (ref)	
	< 3.5 g/dL	1.91 (1.76–2.08)	**8.30E−50**	1.67 (1.50–1.86)	**6.29E−21**
PNI	≥ 44	1 (ref)		1 (ref)	
	< 44	1.70 (1.55–1.86)	**1.37E−30**	1.46 (1.31–1.63)	**2.04E−11**
Overall survival					
Fearon criteria‐based cachexia	No cachexia	1 (ref)		1 (ref)	
	Cachexia	2.27 (2.05–2.51)	**2.65E−58**	1.97 (1.75–2.23)	**6.89E−28**
WLGS	< 2	1 (ref)		1 (ref)	
	≥ 2	2.13 (1.92–2.35)	**1.51E−47**	1.91 (1.69–2.17)	**1.40E−24**
NLR	≤ 3	1 (ref)		1 (ref)	
	> 3	2.43 (2.14–2.77)	**1.84E−41**	1.95 (1.67–2.27)	**3.85E−17**
Albumin	≥ 3.5 g/dL	1 (ref)		1 (ref)	
	< 3.5 g/dL	2.33 (2.10–2.57)	**2.64E−60**	2.09 (1.84–2.36)	**6.06E−31**
PNI	≥ 44	1 (ref)		1 (ref)	
	< 44	2.55 (2.28–2.85)	**7.79E−60**	2.07 (1.80–2.37)	**1.41E−25**

Lung					
Cachexia predictor		Unadjusted HR	*p* value	Adjusted HR	*p* value
Disability‐free survival					
Fearon criteria‐based cachexia	No cachexia	1 (ref)		1 (ref)	
	Cachexia	1.47 (1.25–1.72)	**2.95E−06**	1.41 (1.17–1.70)	**0.0003**
WLGS	< 2	1 (ref)		1 (ref)	
	≥ 2	1.41 (1.20–1.65)	**3.59E−05**	1.38 (1.14–1.66)	**0.00076**
NLR	≤ 3	1 (ref)		1 (ref)	
	> 3	1.50 (1.24–1.83)	**4.73E−05**	1.51 (1.20–1.89)	**0.00049**
Albumin	≥ 3.5 g/dL	1 (ref)		1 (ref)	
	< 3.5 g/dL	1.30 (1.09–1.55)	**0.00419**	1.27 (1.03–1.57)	**0.02402**
PNI	≥ 44	1 (ref)		1 (ref)	
	< 44	1.66 (1.40–1.96)	**2.26E−09**	1.48 (1.22–1.80)	**8.59E−05**
Hospitalization‐free survival					
Fearon criteria‐based cachexia	No cachexia	1 (ref)		1 (ref)	
	Cachexia	1.41 (1.24–1.61)	**1.32E−07**	1.34 (1.15–1.56)	**0.00014**
WLGS	< 2	1 (ref)		1 (ref)	
	≥ 2	1.33 (1.17–1.51)	**1.77E−05**	1.27 (1.10–1.48)	**0.0015**
NLR	≤ 3	1 (ref)		1 (ref)	
	> 3	1.33 (1.13–1.56)	**0.0006**	1.22 (1.02–1.47)	**0.034**
Albumin	≥ 3.5 g/dL	1 (ref)		1 (ref)	
	< 3.5 g/dL	1.78 (1.55–2.04)	**1.25E−16**	1.61 (1.37–1.89)	**7.33E−09**
PNI	≥ 44	1 (ref)		1 (ref)	
	< 44	1.58 (1.38–1.82)	**1.09E−10**	1.42 (1.21–1.67)	**2.53E−05**
Overall survival					
Fearon criteria‐based cachexia	No cachexia	1 (ref)		1 (ref)	
	Cachexia	1.82 (1.57–2.10)	**1.07E−15**	1.68 (1.42–2.00)	**1.69E−09**
WLGS	< 2	1 (ref)		1 (ref)	
	≥ 2	1.67 (1.44–1.94)	**2.33E−11**	1.62 (1.37–1.93)	**4.09E−08**
NLR	≤ 3	1 (ref)		1 (ref)	
	> 3	1.80 (1.48–2.20)	**6.06E−09**	1.72 (1.37–2.17)	**4.29E−06**
Albumin	≥ 3.5 g/dL	1 (ref)		1 (ref)	
	< 3.5 g/dL	1.95 (1.67–2.26)	**7.31E−18**	1.90 (1.59–2.27)	**9.06E−13**
PNI	≥ 44	1 (ref)		1 (ref)	
	< 44	1.91 (1.63–2.25)	**1.84E−15**	1.73 (1.44–2.08)	**8.28E−09**

Renal					
Cachexia predictor		Unadjusted HR	*p* value	Adjusted HR	*p* value
Disability‐free survival					
Fearon criteria‐based cachexia	No cachexia	1 (ref)		1 (ref)	
	Cachexia	1.98 (1.41–2.78)	**8.45E−05**	1.73 (1.14–2.61)	**0.00961**
WLGS	< 2	1 (ref)		1 (ref)	
	≥ 2	1.91 (1.37–2.67)	**0.00014**	1.52 (1.03–2.25)	**0.03641**
NLR	≤ 3	1 (ref)		1 (ref)	
	> 3	2.60 (1.77–3.81)	**1.05E−06**	1.92 (1.23–2.98)	**0.00395**
Albumin	≥ 3.5 g/dL	1 (ref)		1 (ref)	
	< 3.5 g/dL	1.69 (1.20–2.38)	**0.00288**	1.22 (0.78–1.90)	0.38982
PNI	≥ 44	1 (ref)		1 (ref)	
	< 44	2.41 (1.68–3.47)	**2.05E−06**	1.40 (0.89–2.20)	0.14274
Hospitalization‐free survival					
Fearon criteria‐based cachexia	No cachexia	1 (ref)		1 (ref)	
	Cachexia	1.92 (1.47–2.51)	**1.87E−06**	1.72 (1.23–2.41)	**0.00146**
WLGS	< 2	1 (ref)		1 (ref)	
	≥ 2	1.72 (1.32–2.24)	**5.93E−05**	1.44 (1.05–1.99)	**0.02576**
NLR	≤ 3	1 (ref)		1 (ref)	
	> 3	2.29 (1.68–3.13)	**2.00E−07**	1.44 (1.00–2.07)	**0.04983**
Albumin	≥ 3.5 g/dL	1 (ref)		1 (ref)	
	< 3.5 g/dL	2.06 (1.56–2.70)	**2.33E−07**	1.84 (1.26–2.68)	**0.00149**
PNI	≥ 44	1 (ref)		1 (ref)	
	< 44	1.70 (1.26–2.31)	**0.00053**	1.13 (0.77–1.66)	0.53104
Overall survival					
Fearon criteria‐based cachexia	No cachexia	1 (ref)		1 (ref)	
	Cachexia	2.70 (1.92–3.79)	**1.15E−08**	2.25 (1.46–3.49)	**0.00027**
WLGS	< 2	1 (ref)		1 (ref)	
	≥ 2	2.48 (1.75–3.51)	**2.88E−07**	2.08 (1.34–3.22)	**0.00111**
NLR	≤ 3	1 (ref)		1 (ref)	
	> 3	3.20 (2.07–4.96)	**1.93E−07**	2.10 (1.26–3.48)	**0.0042**
Albumin	≥ 3.5 g/dL	1 (ref)		1 (ref)	
	< 3.5 g/dL	2.64 (1.87–3.73)	**3.45E−08**	1.80 (1.13–2.87)	**0.01315**
PNI	≥ 44	1 (ref)		1 (ref)	
	< 44	3.08 (2.08–4.57)	**2.07E−08**	1.66 (1.01–2.75)	**0.04719**

Melanoma					
Cachexia predictor		Unadjusted HR	*p* value	Adjusted HR	*p* value
Disability‐free survival					
Fearon criteria‐based cachexia	No cachexia	1 (ref)		1 (ref)	
	Cachexia	2.31 (1.59–3.35)	**1.22E−05**	1.30 (0.78–2.18)	0.31109
WLGS	< 2	1 (ref)		1 (ref)	
	≥ 2	1.72 (1.21–2.44)	**0.00237**	1.01 (0.64–1.61)	0.95739
NLR	≤ 3	1 (ref)		1 (ref)	
	> 3	2.16 (1.48–3.16)	**6.05E−05**	1.55 (0.95–2.54)	0.07976
Albumin	≥ 3.5 g/dL	1 (ref)		1 (ref)	
	< 3.5 g/dL	2.18 (1.50–3.19)	**5.11E−05**	1.20 (0.70–2.05)	0.50408
PNI	≥ 44	1 (ref)		1 (ref)	
	< 44	2.84 (1.91–4.21)	**2.15E−07**	1.34 (0.74–2.42)	0.3275
Hospitalization‐free survival					
Fearon criteria‐based cachexia	No cachexia	1 (ref)		1 (ref)	
	Cachexia	2.28 (1.77–2.95)	**2.63E−10**	1.87 (1.35–2.58)	**0.00017**
WLGS	< 2	1 (ref)		1 (ref)	
	≥ 2	1.89 (1.50–2.39)	**9.85E−08**	1.47 (1.10–1.97)	**0.0091**
NLR	≤ 3	1 (ref)		1 (ref)	
	> 3	1.76 (1.35–2.31)	**3.66E−05**	1.34 (0.94–1.89)	0.10274
Albumin	≥ 3.5 g/dL	1 (ref)		1 (ref)	
	< 3.5 g/dL	2.12 (1.65–2.73)	**5.27E−09**	1.38 (0.98–1.94)	0.06767
PNI	≥ 44	1 (ref)		1 (ref)	
	< 44	2.08 (1.55–2.79)	**9.75E−07**	1.55 (1.03–2.33)	**0.03402**
Overall survival					
Fearon criteria‐based cachexia	No cachexia	1 (ref)		1 (ref)	
	Cachexia	2.93 (2.08–4.12)	**8.34E−10**	2.56 (1.64–3.98)	**3.29E−05**
WLGS	< 2	1 (ref)		1 (ref)	
	≥ 2	2.33 (1.68–3.24)	**4.55E−07**	1.98 (1.31–2.97)	**0.00108**
NLR	≤ 3	1 (ref)		1 (ref)	
	> 3	3.00 (1.97–4.59)	**3.67E−07**	2.30 (1.34–3.93)	**0.00241**
Albumin	≥ 3.5 g/dL	1 (ref)		1 (ref)	
	< 3.5 g/dL	3.41 (2.44–4.76)	**5.56E−13**	2.72 (1.73–4.30)	**1.58E−05**
PNI	≥ 44	1 (ref)		1 (ref)	
	< 44	3.69 (2.46–5.55)	**3.45E−10**	3.85 (2.12–6.99)	**1.01E−05**

*Note:* Significant *p* values are bolded.

Similar results were seen in lung cancer in adjusted models (Table 3). HRs for cachexia were 1.41 (95% CI 1.17–1.70), 1.34 (95% CI 1.15–1.56) and 1.68 (95% CI 1.42–2.00) for shorter disability‐free, hospitalization‐free and overall survival, respectively. HRs for WLGS ≥ 2 were 1.38 (95% CI 1.14–1.66), 1.27 (95% CI 1.10–1.48) and 1.62 (95% CI 1.37–1.93) for shorter disability‐free, hospitalization‐free and overall survival, respectively. HRs for NLR > 3 were 1.51 (95% CI 1.20–1.89), 1.22 (95% CI 1.02–1.47) and 1.72 (95% CI 1.37–2.17) for shorter disability‐free, hospitalization‐free and overall survival, respectively. HRs for albumin < 3.5 g/dL were 1.27 (95% CI 1.03–1.57), 1.61 (95% CI 1.37–1.89) and 1.90 (95% CI 1.59–2.27) for shorter disability‐free, hospitalization‐free and overall survival, respectively. HRs for PNI < 44 were 1.48 (95% CI 1.22–1.80), 1.42 (95% CI 1.21–1.67) and 1.73 (95% CI 1.44–2.08) for shorter disability‐free, hospitalization‐free and overall survival, respectively.

Similar results were seen in renal cell cancer (Table 3). HRs for cachexia were 1.73 (95% CI 1.14–2.61), 1.72 (95% CI 1.23–2.41) and 2.25 (95% CI 1.46–3.49) for shorter disability‐free, hospitalization‐free and overall survival, respectively. HRs for WLGS ≥ 2 were 1.52 (95% CI 1.03–2.25), 1.44 (95% CI 1.05–1.99) and 2.08 (95% CI 1.34–3.22) for shorter disability‐free, hospitalization‐free and overall survival, respectively. HRs for NLR > 3 were 1.92 (95% CI 1.23–2.98), 1.44 (95% CI 1.00–2.07) and 2.10 (95% CI 1.26–3.48) for shorter disability‐free, hospitalization‐free and overall survival, respectively. Albumin < 3.5 g/dL was associated with shorter hospitalization‐free and overall survival (HRs: 1.84 (95% CI 1.26–2.68) and 1.80 (95% CI 1.13–2.87), respectively). PNI < 44 was associated with shorter overall survival, HR: 1.66 (95% CI 1.01–2.75).

None of the cachexia variables were associated with disability‐free survival in adjusted models for melanoma. Cachexia, WLGS ≥ 2 and PNI < 44 were associated with shorter hospitalization‐free survival—HRs: 1.87 (95% CI 1.35–2.58), 1.47 (95% CI 1.10–1.97) and 1.55 (95% CI 1.03–2.33), respectively. Cachexia, WLGS ≥ 2, NLR > 3, albumin < 3.5 g/dL and PNI < 44 were all associated with shorter overall survival. HRs were 2.56 (95% CI 1.64–3.98), 1.98 (95% CI 1.31–2.97), 2.30 (95% CI 1.34–3.93), 2.72 (95% CI 1.73–4.30) and 3.85 (95% CI 2.12–6.99), respectively (Table 3).

The assumptions of proportional hazards and no major influential or outlier observations were met for all models.

## 4 Discussion

The objective of this study was to determine the ability for routinely collected cachexia variables to predict the clinical endpoints of overall survival, disability and hospitalization for cancer patients undergoing ICI therapy. Cancer‐associated cachexia is a known phenomenon impacting patient outcomes. Previous reports have described reduced ICI therapy response in cancer patients with cachexia [10, 11, 14, 15, 16, 17, 18, 19, 20, 21, 22, 23, 24]. However, these previous studies have been limited by relatively small cohorts (< 200 patients), have focused primarily on nonsmall cell lung cancer (NSCLC) and frequently included predictors that may not be in common clinical practice. Here, we analysed EHR records from a large cohort of 3285 lung, renal, melanoma and other cancer patients who received ICI therapy to determine how frequently the Fearon consensus criteria, WLGS, NLR, albumin, PNI, CRP and mGPS were collected in routine clinical practice and evaluate their prognostic ability at the time of ICI initiation.

There are four major findings from this study. (1) Of the selected cachexia variables, only the Fearon consensus criteria, WLGS, NLR, albumin and PNI were reliably present as data routinely collected at the time of ICI initiation (baseline). CRP and mGPS had high degrees of baseline missingness in our cohort. (2) We confirmed the literature's findings of worse overall survival in 1266 lung cancer patients with cachexia (consensus criteria). (3) The Fearon consensus criteria, WLGS, NLR, albumin and PNI were predictors of worse disability‐free, hospitalization‐free and overall survival in the overall data set and in lung and renal cell cancer. (4) In melanoma patients, we found that the consensus criteria, WLGS, NLR, albumin and PNI predict worse overall survival, whereas the consensus criteria, WLGS and PNI predict worse hospitalization‐free survival.

Several studies have found that CRP and mGPS are indicators of cachexia [33, 34]. However, in our approach focusing on EHR data reflective of current clinical practice in a large‐scale healthcare system, these variables had high degrees of missingness in our cohort at baseline (time of ICI initiation). In our real‐world clinical dataset, 98.2% of patients were missing CRP results, leading to 98.6% of patients missing mGPS. This high degree of missingness at our site means that despite associations of these predictors with clinical outcomes in the literature, current workflow for clinical care of ICI patients does not include CRP making mGPS difficult to use for clinical prognostication. The Fearon consensus criteria for cachexia, WLGS, NLR, albumin and PNI were readily determined using routine clinical measurements and lab tests—weight, height, ANC, ALC and albumin collected as part of vitals, blood count and metabolic panels. Therefore, these features are more readily available for use as clinical prognosticators at the time of ICI initiation.

The majority of previous studies on the associations between cachexia and clinical outcomes in lung cancer patients undergoing ICI therapy found the weight‐based definition of cachexia to be associated with worse overall survival [11, 14, 16, 18, 19, 20, 22, 23]. Morimoto et al. [15] and Murata et al. [21] did not find a significant association, and it is possible that their cohort sizes of 196 and 100 lung cancer patients, respectively, were not sufficiently powered to detect differences between cachexia strata. In our cohort of 1266 lung cancer patients, we indeed found that the Fearon consensus criteria was associated with worse overall survival. This was consistent in 1‐ and 3‐year survival rates and in adjusted Cox models.

In lung and renal cell cancer patients, we found that the Fearon consensus criteria, WLGS, NLR, albumin and PNI generally predicted worse disability‐free, hospitalization‐free and overall survival. Albumin and PNI were not associated with shorter disability‐free survival, and PNI was not associated with shorter hospitalization‐free survival in renal cell cancer. A previous study on renal cell cancer found that NLR was not a significant predictor of overall survival [17], but it may have also been underpowered for this predictor with a cohort size of 52 patients. In our adjusted Cox models for lung cancer, HRs for cachexia were 1.41 (95% CI 1.17–1.70), 1.34 (95% CI 1.15–1.56) and 1.68 (95% CI 1.42–2.00) for shorter disability‐free, hospitalization‐free and overall survival, respectively. We observed similar results in adjusted Cox models showing that WLGS ≥ 2, NLR > 3, albumin < 3.5 g/dL and PNI < 44 were associated with a shorter time to all outcomes studied. Furthermore, we were able to recapitulate these findings from Cox models in a cohort of patients with renal cell cancer. Although previous studies of cachexia in cancer use overall survival as the primary clinical outcome, to our knowledge, this is the first study examining the effect of cachexia on disability and hospitalization rates. Disability, measured by need for rehabilitation services, indicates a clinically significant functional impairment and is an important descriptor of the patient's quality of life and likelihood of maintaining social and functional independence [27]. Hospitalization can be a proxy for worsening of the cancer itself but may also indicate development of other severe adverse events, which are of special interest in the context of ICI therapy [37].

In melanoma, the Fearon consensus criteria, WLGS, NLR, albumin and PNI all predicted worse overall survival. None of the cachexia variables were significant predictors of disability rate, and neither NLR nor albumin was a significant predictor of admission rate. For disability‐free survival, in our adjusted models, ECOG > 0, cancer stage IV and age ≥ 65 years were the significant predictors of disability. In the adjusted model for hospitalization‐free survival for both NLR and albumin, CTLA‐4 checkpoint therapy, ECOG > 0 and cancer stage IV were the significant predictors of hospitalization. It is possible our cohort size for melanoma patients (*N* = 477) was not sufficiently powered to find these associations. Alternatively, prior studies suggest that melanoma patients being given ICI treatment have relatively higher physical function and quality of life compared to other cancer types [38]. Further investigation using alternative outcome measures should be done in a larger cohort of melanoma patients to determine if the Fearon consensus criteria, WLGS, NLR, albumin and PNI may actually associate with physical function and morbidity.

Our study has several limitations. The high degree of missingness of CRP measurements prevented us from determining association of mGPS with survival‐based outcomes, despite the relatively large cohort of 3285 patients. CRP may not be routinely tested for cancer patients receiving ICI therapy at our site; therefore, CRP and mGPS may be less useful as predictors for clinical decision making in this population. Another limitation is that this is a single‐site study. However, our findings corroborate what previous studies have found with the Fearon consensus criteria for cachexia being a predictor of worse overall survival in lung cancer. This increases confidence in the generalizability of our findings that the Fearon consensus criteria, WLGS, NLR, albumin and PNI predict worse overall survival, time to disability and time to emergency or inpatient admission in lung cancer, renal cell cancer and melanoma.

Our findings suggest several avenues for future studies. The cachexia predictors—the Fearon consensus criteria for cachexia, WLGS, NLR, albumin and PNI—demonstrated clear negative association with overall survival as well as the functional outcomes of disability‐free and hospitalization‐free survival. As these predictors are readily available in clinical practice, they could serve as indications for interventions such as prehabilitation or nutrition impact symptom directed therapy for high‐risk patients in preparation for ICI therapy initiation. Indeed, in our overall cohort, we saw only a mild increase in likelihood for patients with any one of the Fearon criteria, WLGS, NLR, albumin or PNI markers to have a dietitian visit after initiating ICI therapy (Supporting Information Results and Table 3). Given that most oncology providers do not actively incorporate these markers into their decision making, prospective studies could work to improve the implantation of these markers and thereby improve the likelihood that patients will get appropriate standard care for addressing nutrition impact symptoms while undergoing ICI therapy. In addition, use of these markers will encourage the inclusion of the ICI therapy population in clinical trials involving developmental therapeutics for cachexia.

In summary, we found five cachexia variables—the Fearon consensus criteria for cachexia, WLGS, NLR, albumin and PNI—to be routinely collected at the time of ICI initiation in regular clinical practice and predictive of worse disability‐free, hospitalization‐free and overall survival in our cohort of all patients receiving ICI therapy. All five cachexia variables predict worse overall survival, shorter time to disability and shorter time to admission in lung and renal cell cancer patients and worse overall survival in melanoma patients receiving ICI therapy. As these variables can be calculated from routinely collected clinical data points—patient weight, height, ANC, ALC and albumin—these findings may be useful for clinical prognostication and decision making around cachexia interventions.

## Ethics Statement

This study was governed by the Northwestern University Institutional Review Board (NU IRB), Protocol Nos. STU00210502 and STU00206779. We received a waiver of consent from the NU IRB to analyse the data from this cohort given impracticality of consent and low risk of the study.

## Conflicts of Interest

Dr. Walunas receives unrelated research funding from Gilead Sciences.

## Supporting information


**Figure S1** Kaplan–Meier survival curves in lung cancer for the cachexia predictors – the Fearon consensus criteria for cachexia, weight loss grading system (WLGS), neutrophil to lymphocyte ratio (NLR), albumin, and the prognostic nutritional index (PNI) – compared to the clinical endpoints – overall survival, time to rehabilitation order, and time to emergency or inpatient admission. P‐values were calculated by log‐rank test.
**Figure S2** Kaplan–Meier survival curves in renal cell cancer for the cachexia predictors – the Fearon consensus criteria for cachexia, weight loss grading system (WLGS), neutrophil to lymphocyte ratio (NLR), albumin, and the prognostic nutritional index (PNI) – compared to the clinical endpoints – overall survival, time to rehabilitation order, and time to emergency or inpatient admission. P‐values were calculated by log‐rank test.
**Figure S3** Kaplan–Meier survival curves in melanoma for the cachexia predictors – the Fearon consensus criteria for cachexia, weight loss grading system (WLGS), neutrophil to lymphocyte ratio (NLR), albumin, and the prognostic nutritional index (PNI) – compared to the clinical endpoints – overall survival, time to rehabilitation order, and time to emergency or inpatient admission. P‐values were calculated by log‐rank test.
**Table S1** Cancer and ICI electronic health record data definitions.
**Table S2** Regular expression search strings to extract cancer related variables from clinical notes.
**Table S3** Odds ratios for having a nutritionist or dietitian visit within 30 days after initiating ICI therapy given each cachexia predictor for the overall cohort. Signficant p‐values are bolded.
