# Supplementary material for: Weight and Blood‐Based Markers of Cachexia Predict Disability, Hospitalization and Worse Survival in Cancer Immunotherapy Patients
Source: J Cachexia Sarcopenia Muscle. 2025 Jan 16;16(1):e13685. doi: 10.1002/jcsm.13685 (PMC11736629; doi:10.1002/jcsm.13685)
Supplement: Supplementary file 1 — Figure S1 Kaplan–Meier survival curves in lung cancer for the cachexia predictors – the Fearon consensus criteria for cachexia, weight loss grading system (WLGS), neutrophil to lymphocyte ratio (NLR), albumin, and the prognostic nutritional index (PNI) – compared to the clinical endpoints – overall survival, time to rehabilitation order, and time to emergency or inpatient admission. P‐values were calculated by log‐rank test. Figure S2 Kaplan–Meier survival curves in renal cell cancer for the cachexia predictors – the Fearon consensus criteria for cachexia, weight loss grading system (WLGS), neutrophil to lymphocyte ratio (NLR), albumin, and the prognostic nutritional index (PNI) – compared to the clinical endpoints – overall survival, time to rehabilitation order, and time to emergency or inpatient admission. P‐values were calculated by log‐rank test. Figure S3 Kaplan–Meier survival curves in melanoma for the cachexia predictors – the Fearon consensus criteria for cachexia, weight loss grading system (WLGS), neutrophil to lymphocyte ratio (NLR), albumin, and the prognostic nutritional index (PNI) – compared to the clinical endpoints – overall survival, time to rehabilitation order, and time to emergency or inpatient admission. P‐values were calculated by log‐rank test. Table S1 Cancer and ICI electronic health record data definitions. Table S2 Regular expression search strings to extract cancer related variables from clinical notes. Table S3 Odds ratios for having a nutritionist or dietitian visit within 30 days after initiating ICI therapy given each cachexia predictor for the overall cohort. Signficant p‐values are bolded. [file JCSM-16-e13685-s001.docx]

SUPPLEMENTAL METHODS AND RESULTS

Association of cachexia predictors with nutritionist or dietitian visits

We evaluated nutritionist or dietitian visits, extracted from the Northwestern Medicine Enterprise Data Warehouse (NMEDW), within 30 days after ICI therapy initiation as a possible cachexia intervention. Association between dichotomized cachexia predictors (cachexia (Fearon consensus criteria, no-cachexia as reference), WLGS ≥2 (WLGS <2 reference), NLR >3 (NLR ≤3 reference), albumin <3.5 g/dL (albumin ≥3.5 g/dL reference), and PNI <44 (PNI ≥44 reference)) and nutritionist/dietitian visits were determined in the overall cancer/ICI cohort by Fisher Exact test.

Patients with positive cachexia predictors were more likely to see a nutritionist or dietitian

Patients with cachexia (Fearon consensus criteria, no-cachexia as reference), WLGS ≥2 (WLGS <2 reference), NLR >3 (NLR ≤3 reference), albumin <3.5 g/dL (albumin ≥3.5 g/dL reference), and PNI <44 (PNI ≥44 reference) were all more likely to have a nutritionist or dietitian visit within 30 days after initiating ICI therapy. ORs for nutritionist or dietitian visit within 30 days after ICI therapy initiation: 1.80 (95% CI 1.28-2.55), 1.69 (95% CI 1.24-2.30), 1.66 (95% CI 1.14-2.47), 1.49 (95% CI 1.09-2.02), and 1.66 (95% CI 1.19-2.34), respectively.

| Supplemental Table 1. Cancer and ICI electronic health record data definitions | |
| --- | --- |
| Cancer/Immunotherapy | Codes |
| Melanoma | ICD-9-CM: 172 ICD-10-CM: C43 |
| Renal cell carcinoma | ICD-9-CM: 189 ICD-10-CM: C64 |
| Lung cancer | ICD-9-CM: 162 ICD-10-CM: C34 |
| Urothelial cancer | ICD-9-CM: 188 ICD-10-CM: C67 |
| Head and neck cancer | ICD-9-CM: 140, 141, 142, 143, 144, 145, 146, 147, 148, 149, 160, 161 ICD-10-CM: C00, C01, C02, C03, C04, C05, C06, C07, C08, C09, C10, C11, C12, C13, C14 |
| Gastric cancer | ICD-9-CM: 151 ICD-10-CM: C16 |
| Colon cancer | ICD-9-CM: 153 ICD-10-CM: C18 |
| Liver cancer | ICD-9-CM: 155 ICD-10-CM: C22 |
| Cervical cancer | ICD-9-CM: 180 ICD-10-CM: C53 |
| Endometrial cancer | ICD-9-CM: 179, 182 ICD-10-CM: C54, C55 |
| Breast cancer | ICD-9-CM: 174, 175 ICD-10-CM: C50 |
| Hodgkins disease | ICD-9-CM: 201 ICD-10-CM: C81 |
| Merkel cell carcinoma | ICD-9-CM: ‘209.31’, ‘209.32’, ‘209.33’, ‘209.34’, ‘209.35’, ‘209.36’ ICD-10-CM: C4A |
| Rectal cancer | ICD-9-CM: 154.1 ICD-10-CM: C20 |
| Prostate cancer | ICD-9-CM: 185 ICD-10-CM: C61 |
| Esophageal cancer | ICD-9-CM: 150 ICD-10-CM: C15 |
| Leukemia | ICD-9-CM: 204, 205, 206, 207, 208 ICD-10-CM: C91, C92, C93, C94, C95 |
| Other lymphoma | ICD-9-CM: 200, 202 ICD-10-CM: C82, C83, C84, C85, C86, C88 |
| Other cancer | ICD-9-CM: 195 ICD-10-CM: C76 |
| Pembrolizumab | Regex: 'pembrolizumab', 'keytruda' |
| Nivolumab | Regex: 'nivolumab', 'opdivo' |
| Cemiplimab | Regex: 'cemiplimab', 'libtayo' |
| Avelumab | Regex: 'avelumab', 'bavencio' |
| Durvalumab | Regex: 'durvalumab', 'imfinzi' |
| Atezolizumab | Regex: 'atezolizumab', 'tecentriq' |
| Ipilimumab | Regex: 'ipilumumab', 'yervoy' |
| Tremilumumab | Regex: 'tremilumumab' |

| Supplemental Table 2. Regular expression search strings to extract cancer related variables from clinical notes | |
| --- | --- |
| Variable | Regular Expression Strings |
| Stage | (?=(?P<extract>(?i).{20}stage.{20}))' '((?i)stage:?\s+[IV]{1,3})[ABCD]?\W' '((?i)stage:?\s+[IV]{1,3})[ABCD][1-5]?\W'  '(?=(?P<extract>(?i).{50}stag[ei].{100}))' '((?i)stage at diagnosis[^\.]{0,5}[\W\s][IV]{1,3})[ABCD]?\W' |
| ECOG | (?=(?P<extract>(?i).{20}ecog.{100}))' '((?i)ecog(?:(?:\s*ps)\|(?:\s*(?:performance\s*)?(?:status\|scale\|grade)))?:?\s+=?-?\s*\(?(?:\d+\|zero\|one\|two\|three\|four\|five)\)?)\W' '((?i)ecog(?:(?:\s*ps)\|(?:\s*(?:performance\s*)?(?:status\|scale\|grade)))?:?\s*=?-?\s*\(?(?:\d+\|zero\|one\|two\|three\|four\|five)\)?)\W' '((?i)ecog\)?(?:(?:\s*ps\)?)\|(?:\s*(?:performance\s*)?(?:status\|scale\|grade)))?:?\s*=?-?\s*\(?(?:\d+\|zero\|one\|two\|three\|four\|five)\)?)' '((?i)ecog(?:(?:\s*ps)\|(?:\s*(?:performance\s*)?(?:status\|scale\|grade)))?:?\s*(?:equivalent\|is\|of\|remains\|(?:\(kps for cns\):?))\s*\(?(?:\d+\|zero\|one\|two\|three\|four\|five)\)?)' '((?i)ecog\)?(?:(?:\s*ps\)?)\|(?:\s*(?:performance\s*)?(?:status\|scale\|grade)))?:?_*\(?(?:\d+\|zero\|one\|two\|three\|four\|five)\)?)'  '(?=(?P<extract>(?i).{50}performance.{100}))' '((?i)(?:performance\s*)(?:status\|scale\|grade)\s*:?\s*[=-–]?\s*\(?(?:\d+\|zero\|one\|two\|three\|four\|five)\)?)\W'  '(?=(?P<extract>(?i).{50}\Wps[\d\W].{100}))' '((?i)(?:ps)\W{0,4}\(?(?:\d+\|zero\|one\|two\|three\|four\|five)\)?)' |


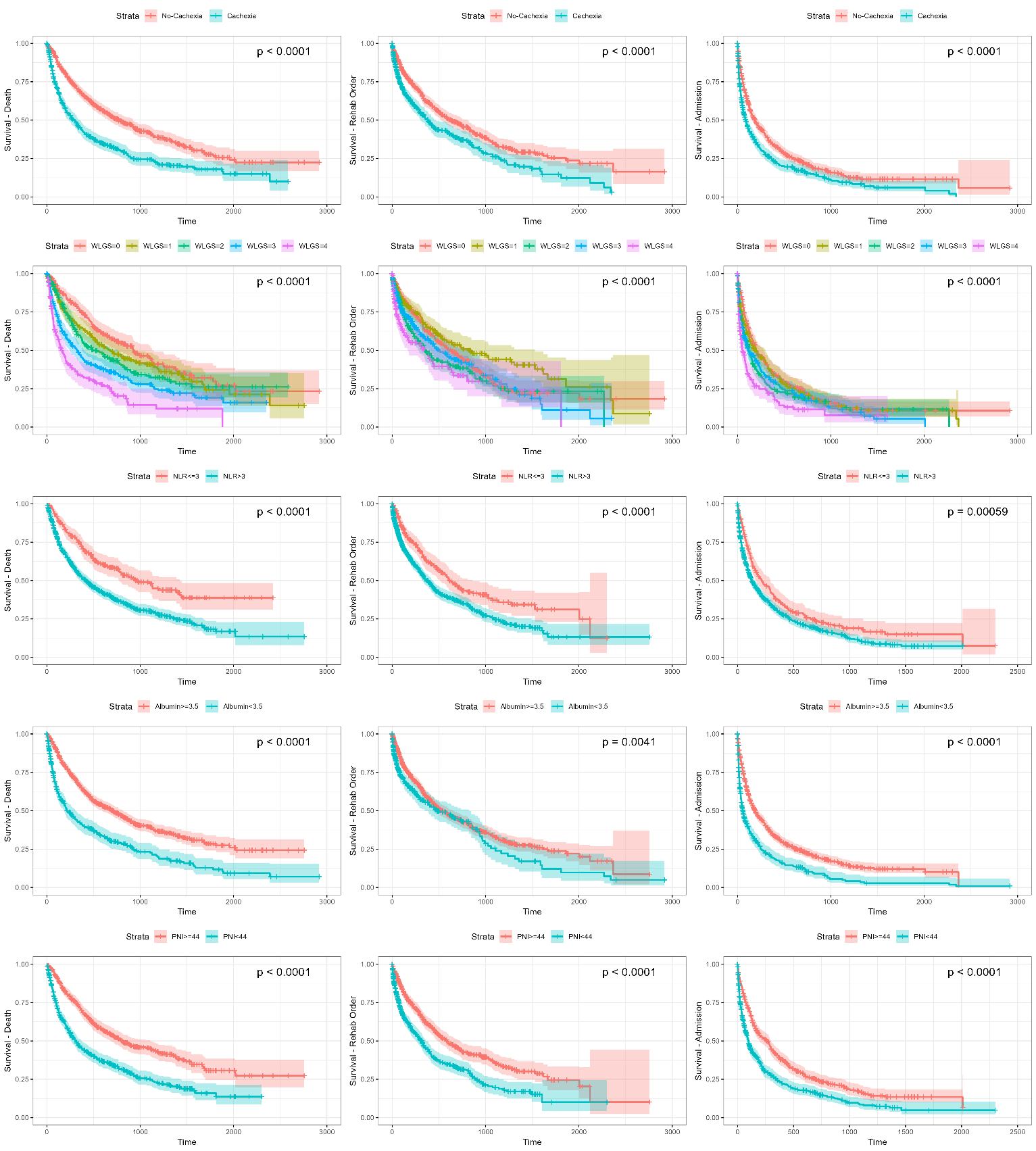


Supplemental Figure 1. Kaplan-Meier survival curves in lung cancer for the cachexia predictors – the Fearon consensus criteria for cachexia, weight loss grading system (WLGS), neutrophil to lymphocyte ratio (NLR), albumin, and the prognostic nutritional index (PNI) – compared to the clinical endpoints – overall survival, time to rehabilitation order, and time to emergency or inpatient admission. P-values were calculated by log-rank test.


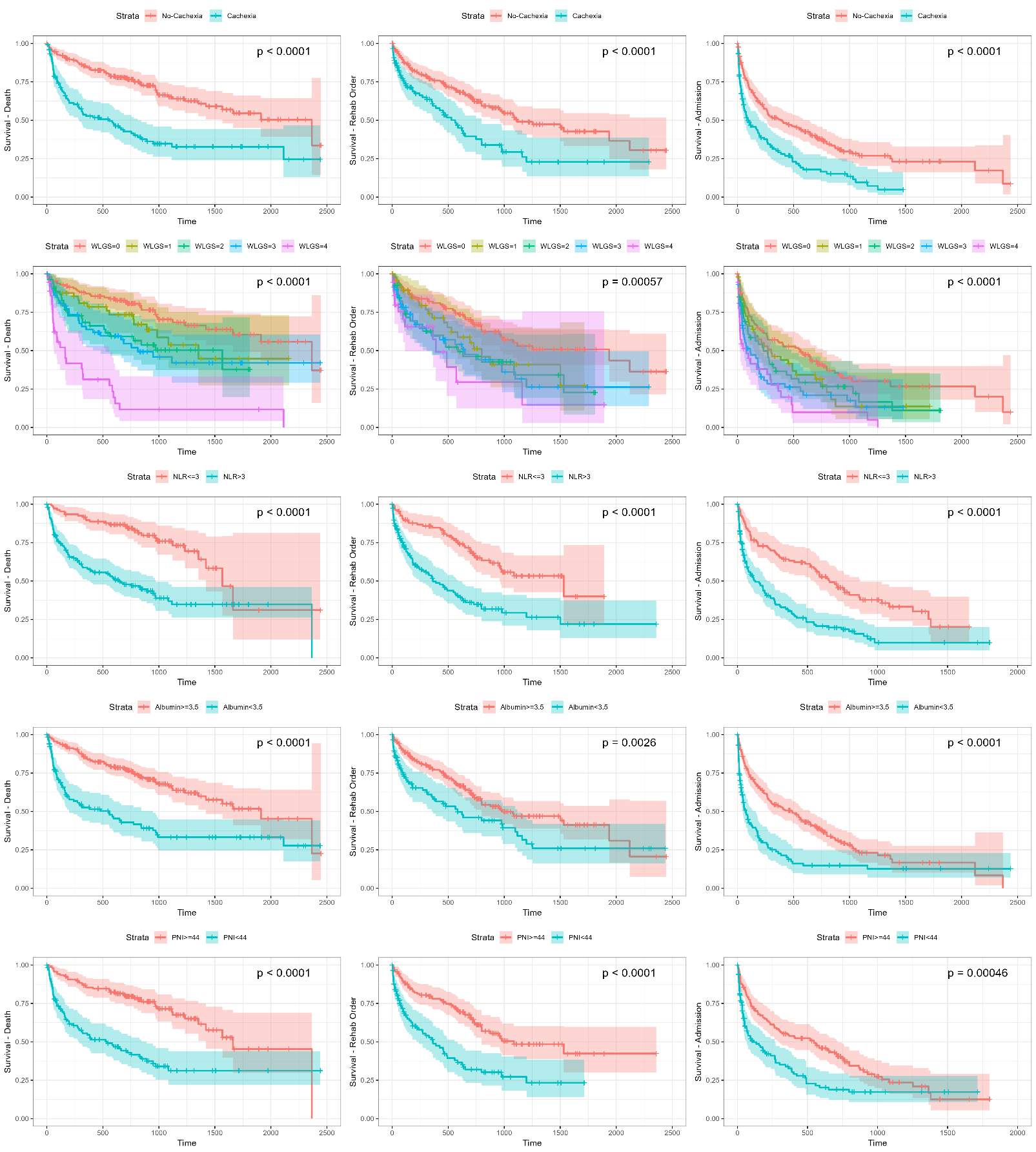


Supplemental Figure 2. Kaplan-Meier survival curves in renal cell cancer for the cachexia predictors – the Fearon consensus criteria for cachexia, weight loss grading system (WLGS), neutrophil to lymphocyte ratio (NLR), albumin, and the prognostic nutritional index (PNI) – compared to the clinical endpoints – overall survival, time to rehabilitation order, and time to emergency or inpatient admission. P-values were calculated by log-rank test.


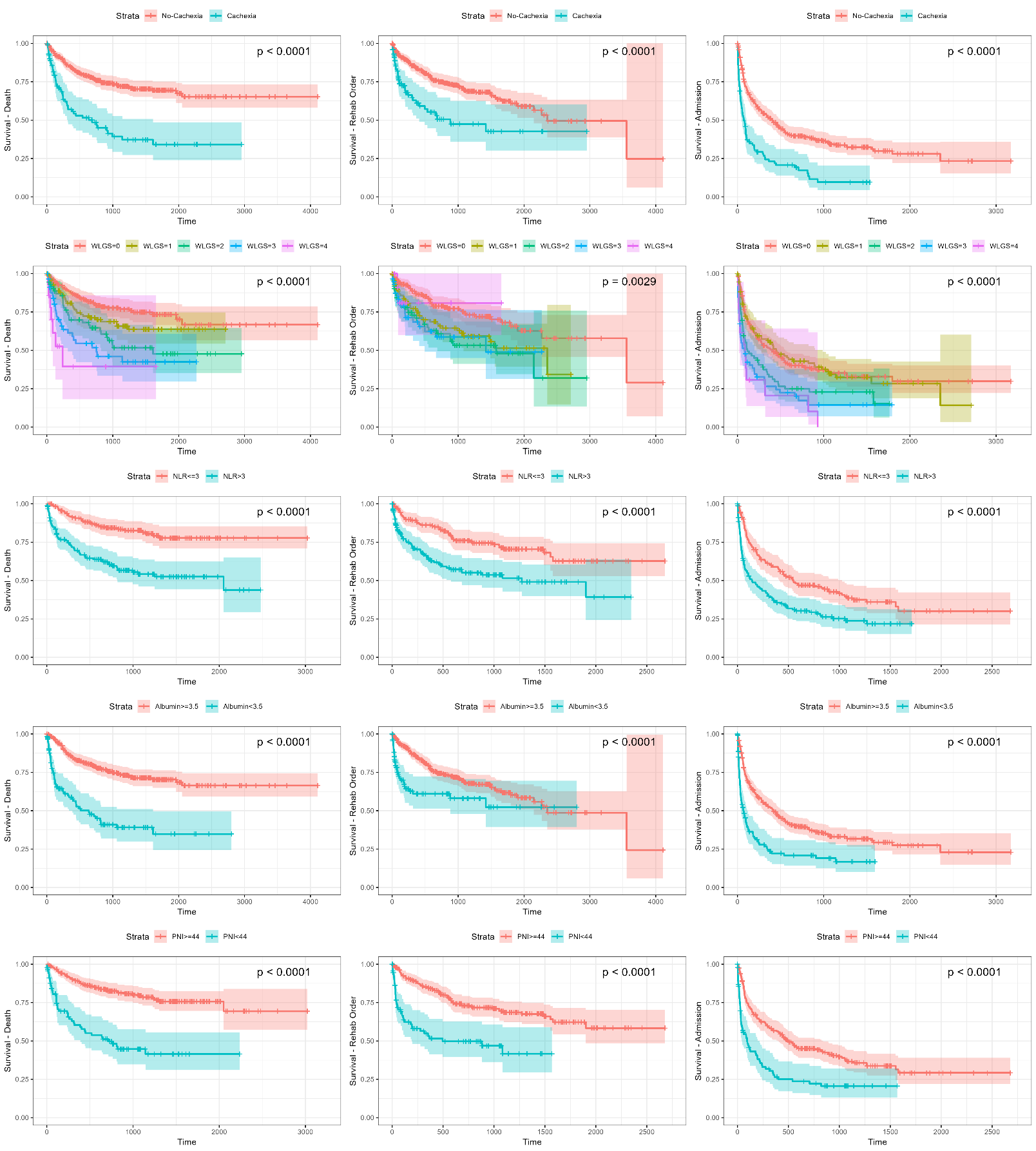


Supplemental Figure 3. Kaplan-Meier survival curves in melanoma for the cachexia predictors – the Fearon consensus criteria for cachexia, weight loss grading system (WLGS), neutrophil to lymphocyte ratio (NLR), albumin, and the prognostic nutritional index (PNI) – compared to the clinical endpoints – overall survival, time to rehabilitation order, and time to emergency or inpatient admission. P-values were calculated by log-rank test.

| Supplemental Table 3. Odds ratios for having a nutritionist or dietitian visit within 30 days after initiating ICI therapy given each cachexia predictor for the overall cohort.  Significant p-values are bolded. | | | |
| --- | --- | --- | --- |
| Cachexia Predictor |  | Odds Ratio | p-value |
| 30 days post ICI therapy initiation | | | |
| Fearon criteria-based cachexia | No cachexia | 1 (ref) |  |
|  | Cachexia | 1.80 (1.28-2.55) | 5.89E-06 |
| WLGS | <2 | 1 (ref) |  |
|  | >=2 | 1.69 (1.24-2.30) | 0.00052866 |
| NLR | <=3 | 1 (ref) |  |
|  | >3 | 1.66 (1.14-2.47) | 0.00633834 |
| Albumin | >=3.5 | 1 (ref) |  |
|  | <3.5 | 1.49 (1.09-2.02) | 0.00941996 |
| PNI | >=44 | 1 (ref) |  |
|  | <44 | 1.66 (1.19-2.34) | 0.00217262 |
